# Methaemoglobin and COHb in patients with malaria

**DOI:** 10.1186/1475-2875-13-285

**Published:** 2014-07-23

**Authors:** Thomas Hänscheid, Tom Gresnigt, Sascha Löhr, Arnaud Flamen, Thomas Zoller, José Melo-Cristino, Martin P Grobusch

**Affiliations:** 1 Centre de Recherches Médicales en Lambaréné (CERMEL), Hôpital Albert Schweitzer, Lambaréné, Gabon; 2 Instituto de Microbiologia, Faculdade de Medicina, Lisbon, Portugal; 3 Center for Tropical Medicine and Travel Medicine, Department of Infectious Diseases, Division of Internal Medicine, Academic Medical Center, University of Amsterdam, Meibergdreef 9, PO Box 22660, 1100 DD Amsterdam, the Netherlands; 4 Charité Universitätsmedizin, Berlin, Germany; 5 Serviço de Patologia Clínica, Centro Hospitalar Lisboa Norte, Lisbon, Portugal; 6 Institute of Tropical Medicine, University of Tübingen, Tübingen, Germany

## Abstract

**Background:**

Haemolytic conditions may contribute to disease pathogenesis and severe clinical manifestations through the liberation of free haemoglobin (Hb) and production of toxic free haem. Thus, free Hb and haem should be associated with altered MetHb and COHb levels in malaria as in other conditions.

**Methods:**

This study comprises data collected at three different sites: (i) a retrospective analysis of the first arterial blood gas result (ABGS) of any patient during 2010 at the University Hospital in Lisbon; (ii) a retrospective analysis of ABGS from patients with severe malaria admitted to the intensive care unit in Berlin, Germany; and (iii) a prospective study of non-invasive MetHb measurements in children with and without malaria in Lambaréné, Gabon.

**Results:**

In Lisbon, the mean MetHb level was 1.4% (SD: 0.5) in a total of 17,834 ABGS. Only 11 of 98 samples with a MetHb level of >3.0 referred to infections. COHb levels showed no particular association with clinical conditions, including sepsis. In 13 patients with severe malaria in Berlin, the mean MetHb levels on admission was 1.29%, with 1.36% for cerebral malaria and 1.14% for non-cerebral malaria (P > 0.05). All COHb measurements were below 2.3%. In Lambaréné, Gabon, 132 healthy children had a mean MetHb level of 1.57%, as compared to 150 children with malaria, with a value of 1.77% and 2.05% in uncomplicated and complicated cases, respectively (P < 0.01).

**Conclusions:**

The data appears consistent with the methaemoglobin/haem hypothesis in malaria and sepsis pathogenesis. However, although MetHb was significantly different between healthy controls and children with malaria in Africa, the difference was rather small, also when compared to previous studies. Still, non-invasive bedside MetHb testing may warrant further evaluation as it could be a simple adjuvant tool for prognosis in resource poor settings.

## Background

Conditions which cause haemolysis and lead to free haemoglobin (Hb) appear to contribute to disease pathogenesis and often severe clinical manifestations, such as renal impairment, vascular disease or inflammation [1]. One proposed mechanism is the liberation of haem from Hb with its pro-inflammatory effects [2,3]. The proposed chain of events starts with the lysis of red blood cells (RBC) and the production of free haemoglobin (Hb) which is oxidized to methaemoglobin (MetHb), liberating the haem group [4]. Haem oxygenase (HO-1) degrades haem, producing biliverdin, iron and carbon monoxide (CO) [5] and subsequently carboxyhaemoglobin (COHb) [6]. While it has been suggested that the liberation of haem is pro-inflammatory and thus contributes to severe forms of infections, such as sepsis or malaria, HO-1 and CO are postulated to have protective effects, as hinted at by the beneficial effects observed after administration of CO in mouse models of these conditions [7,8]. In this context it is noteworthy that increased HO-1 expression in man was reported in fatal falciparum malaria and sepsis [9]. Free haem is not limited to infections and has also been associated with non-infectious haemolytic conditions, such as sickle cell disease [10].

Consequently, one would expect that the presence of free Hb and haem, as well as the increase of HO-1 should be associated with altered MetHb and COHb levels in malaria and possibly other severe infections. Indeed, in an experimental cerebral malaria model in rodents, MetHb levels were significantly raised as compared to non-infected controls [7]. Although unexpectedly, no differences were reported for COHb levels [11].

Patients with overt haemolysis as in sickle cell crisis often have noticeably increased MetHb and COHb levels [12,13]. Three studies in malaria reported different MetHb levels as compared to controls, although the magnitude of the difference as well as the observed values varied several-fold between studies [14-16]. The picture for COHb appears even more complex. Markedly raised levels were reported in Kenyan children and Indonesian adults, with mean values in the order of 4-5% and 7-10%, respectively; two to three-fold the control value [16,17]. Puzzlingly, in Kenya, all children admitted to hospital had raised COHb levels, independent of their underlying disease [17]. As pointed out in these studies, COHb levels may be strongly influenced by external sources of CO, for example cooking fires or cigarette smoke, while MetHb levels ought to reflect closer the underlying haemolytic process [4].

**Figure 1:**
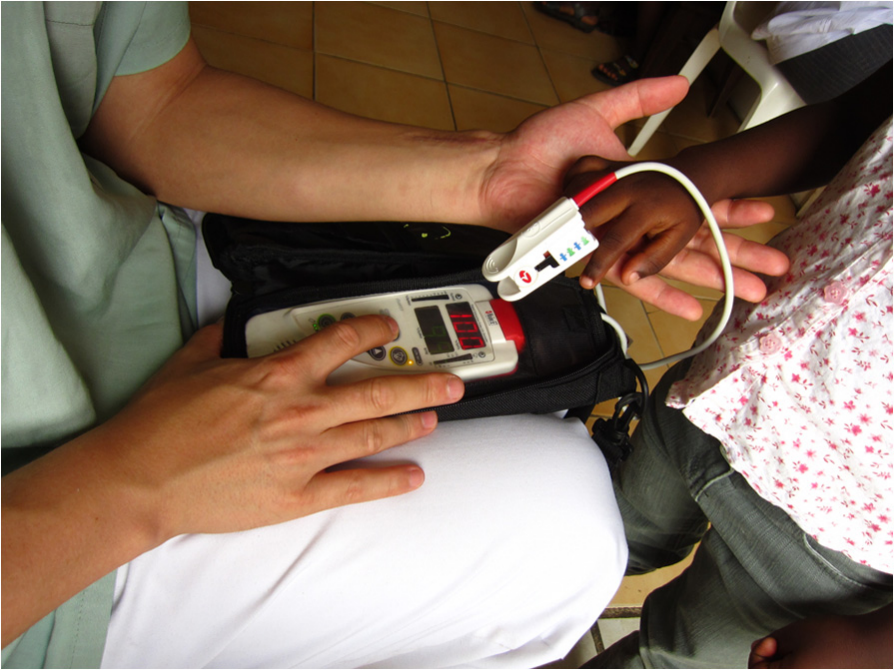
**Methaemoglobin measurement with the pulse oximeter.** RAD-57™ “Rainbow”® Pulse oximeter (Masimo) with fingerprobe for MetHb detection applied to the third finger of the right hand of a child (healthy control) during measurement. The finger/hand is held to reduce movement for 45–60 seconds and the procedure is non-invasive and painless.

MetHb and COHb are usually invasive tests, which require a blood sample. In affluent countries they are determined as part of the arterial blood gas analysis (ABGS), using sophisticated instruments, often not available in malaria endemic areas. A new type of pulse oximeter, the RAD-57™ (Masimo Inc., Irvine/CA/USA) allows to measure MetHb non-invasively (Figure 1) if appropriate finger probes are used [18]. Thus, the usefulness of measuring haemoglobin subtypes in malaria can be revisited.

The objective of the study was to establish values for MetHb and COHb, measured invasively, in (i) a general patient population of a European tertiary care hospital (Lisbon) with or without evidence of infection and/or sepsis and (ii) in a retrospective cohort of patients with severe malaria in Europe (Berlin). The Lisbon data served to establish baseline values for MetHb and COHb, while the Berlin study served to determine which would be best parameters to be measured in Gabon, based on the observed alterations in severe malaria. Based on these results, (iii) the study prospectively investigated the usefulness of non-invasive measurement of MetHb in Gabonese children with malaria.

## Methods

This study comprises data collected during three studies at three different sites: (i) a retrospective analysis of all arterial blood gas results performed during 2010 at the University Hospital in Lisbon and for which MetHb and COHb was also measured, (ii) a retrospective analysis of ABGS from patients admitted to the intensive care unit at Charité University Medical Center in Berlin, Germany, with severe malaria in the years from 2003 to 2007, and (iii) a prospective study of MetHb measurements in children with and without malaria at CERMEL/HAS, Lambaréné, Gabon during the year 2012. Each part of the study was approved by the ethics committee at the respective study site in Lisbon, Berlin and Lambaréné. In Lambaréné informed consent was obtained from parents/guardians prior to inclusion.

In Lisbon, data from all those arterial blood gas analyses for which MetHb and COHb results were also available were obtained from the Laboratory Information System (LIS) for a one-year period. Only the first result in a series of results of the same episode was used, thought to represent the result on presentation. Information on the location/department of request and clinical information which accompanied the request were recorded. Arterial blood gases were analysed on a ABL 800 FLEX (Radiometer, Copenhagen, Denmark) as described by the manufacturer. All results were analysed to establish associations between MetHb, COHb and clinical information.

In Berlin, results were retrieved retrospectively for a cohort of patients with severe malaria who had been cared for on the Intensive Care Unit (ICU). MetHb and COHb mean values for the first day on ICU and in the clinical course were analysed for differences between groups. Arterial blood gases were determined on a ABL 800 Flex instrument (Radiometer, Copenhagen).

At the Hospital Albert Schweitzer in Lambaréné (HAS), children suffering either from non-complicated malaria or severe malaria were prospectively included in the study. Control subjects from the local kindergarden of the HAS and a primary school in town were invited to participate. Children with sickle cell disease were excluded. Sickle cell status was determined by electrophoresis. MetHb levels were measured non-invasively using a RAD-57™, pulse oximeter with a pediatric MetHb rainbow® finger probe (Masimo, Irvine CA, USA). The measurement involves placing the finger probe on the third finger and immobilizing the hand during the measurement for about 45–60 seconds (Figure 1). Each measurement was obtained in triplicate and the mean value was calculated for further analysis. Blood samples were only obtained from children with malaria. Haemoglobin values were obtained (ABX pentra 60, Horiba Medical, Montpellier/France) and malaria diagnosis and parasitaemia were established in thick films using the Lambaréné method for reading slides [19].

Data was analysed in SPSS version 15.0 (IBM Corporation, Armonak, NY, USA). Results were analysed using the student t-test for unpaired data, the Chi-Square test or Fisher’s Exact test and differences of p < 0.05 were considered to be statistically significant.

## Results

**Figure 2:**
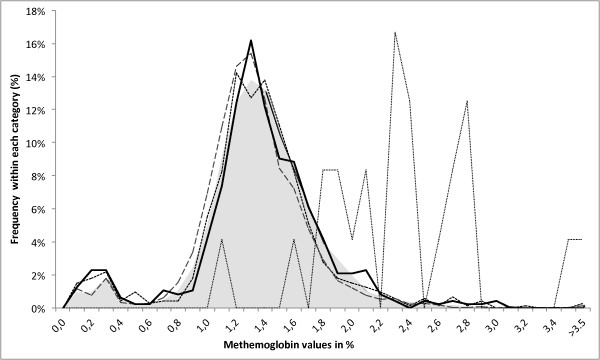
**Methaemoglobin values in an unselected hospital population in Lisbon.** Distribution of methaemoglobin values retrieved retrospectively from the laboratory information system for a one year period from an unselected population at the University Hospital, Lisbon (n = 17,834). Stratified by clinical information accompanying the request into major groups: shaded grey area – no infection (n = 14,062), solid black line – sepsis (n = 476), dashed line - respiratory infection (n = 2,273), square dotted line –other infections (n = 725). As comparison for haemolytic condition: thin dotted line – sickle cell disease (n = 24). Histogram based on 0.1% intervals (bins) of MetHb values. Y-axis represents percentage in each category. No significant difference (p = 0.63) between no infection with a mean of 1.37% and sepsis with a mean of 1.36%.

In Lisbon, 17,834 arterial blood gas results from patients (50% male/50% female) with a mean age of 61.5 years (SD: 22.9, range: 1 week – 103 years) were retrieved. Overall, the mean MetHb level was 1.4% (SD: 0.5, range: 0.1%-13.1%) and the distribution for infectious diseases, including sepsis are shown in Figure 2. The distribution of all infectious categories virtually equals the ‘no infections’ category. Interestingly, there is no statistically significant difference (p = 0.63) between sepsis and absence of infection. Although the number is very low (n = 24), the samples with the clinical information ‘sickle cell disease’ showed higher MetHb levels, as one would expect in such a haemolytic condition (Figure 2, thin dotted line). Of 98 samples with a MetHb level of >3.0 only 11 had infections as per clinical information, eight of them a respiratory tract infection. The correlation between MetHb and haemoglobin (r = -0.18, P > 0.001) or MetHb and COHb (r = -0.03, P < 0.001) was low. COHb levels showed no particular association with clinical conditions, including sepsis. The only exception was, unsurprisingly, CO poisoning/inhalation (Additional file 1).

**Figure 3:**
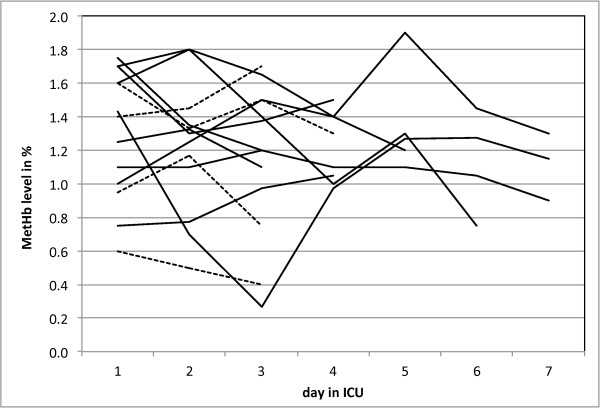
**Methaemoglobin values in patients with complicated malaria admitted to an Intensive Care Unit in Berlin.** Methaemoglobin values retrieved retrospectively from patients admitted with malaria to the Intensive Care Unit at the Charité University Hospital in Berlin (n = 13). All patients had criteria for complicated malaria, with 8 patients having cerebral malaria (lines) and 5 patients having other criteria, as shown in Table 1 (dashed lines). The mean MetHb values was calculated for each day while patients were in the ICU and lengths of the lines corresponds to time in the ICU. Mean value of MetHb levels for the day on admission was 1.29% (SD: 0.38) for all measurements, and 1.36% (SD:0.36) for cerebral malaria while it was 1.14% (SD:0.36) for non-cerebral malaria (NS).

In Berlin, ABGS from 13 patients who fulfilled the WHO criteria for severe malaria admitted to the intensive care unit (ICU) were retrospectively retrieved. The patient’s main characteristics are shown in Table 1, including clinical information, such as haemolysis. The distribution of mean values of MetHb on admission and for the following days in the ICU varied little, as shown in Figure 3. No single MetHb level above 2% was observed (Table 1). The mean value of the MetHb levels for all patients on the day of admission was 1.29% (SD: 0.38); 1.36% (SD: 0.36) for cerebral malaria and 1.14% (SD:0.45) for non-cerebral malaria, with no statistically significant difference. Results for all COHb measurements during their ICU stay are summarized in Additional file 2 which shows that all results were below 2.3%, except for one measurement of 3.7% in a patient with signs of haemolytic anaemia (Table 1).

**Table 1:** Characteristics of patients with severe malaria admitted to the ICU in Berlin

**Age (y)**	***P.f.*** (%)*	**Severe malaria**	**Days (ICU)**	ABGS (n)**	**MetHb % range**	**Remarks**
52	1.0%	CM	4	7	1-1.6	Septic shock
62	0.5%	CM, ARDS, ARF	3	4	1.4-1.7	Pneumonia, sepsis
53	16.0%	CM, ARF, HP	3	5	0.4-0.6	HIV
62	17.0%	CM, ARF, HP	3	7	1.1-1.6	Shock, diarrhoea, UTI
49	8.0%	CM, HP	7	23	0.2-1.5	
39	12.0%	CM, HP	5	7	1.2-1.8	Black water fever, rhabdomyolysis,
39	31.0%	CM, HP, ARF	3	7	0.6-1.3	Pancreatitis
46	10.0%	CM, HP, ARF	4	14	0.7-1.8	HCV, diarrhoea, haemolytic anaemia
59	15.0%	CM, HP, ARF	4	14	0.5-1.3	
29	20.0%	HP	6	14	0.3-1.9	Anaemia
32	7.0%	HP	7	12	0.9-1.2	
60	9.5%	HP	7	17	1-1.9	Anaemia - Hb 6.6,
28	8.0%	HP	3	6	1-1.8	Black water fever

Characteristics of patients admitted to the Intensive Care Unit (ICU) in Berlin with criteria of severe malaria: CM - cerebral malaria, ARF – acute renal failure, ARDS –acute respiratory distress syndrome, HP – hyperparasitaemia. Therapy with either standard intravenous quinine (Q) or artesunate (AS). * = *Plasmodium falciparum* parasitaemia in %; ** = number of measurements of arterial blood gases during ICU stay.

**Figure 4:**
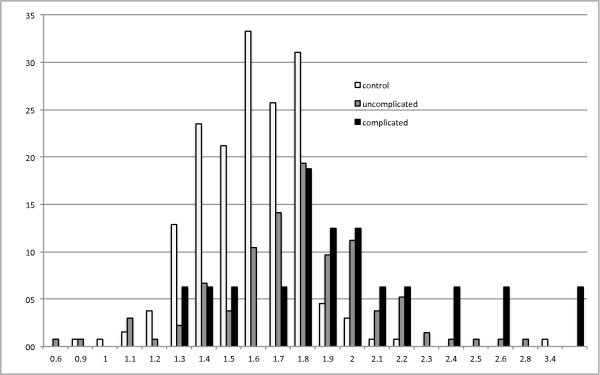
**Methaemoglobin values in children with malaria and healthy controls in Lambaréné, Gabon.** Methemglobin values measured with the pulse oximeter in children in Lambaréné, Gabon (Figure 1). Bars with light upper diagonal pattern show healthy controls (n = 132), grey bars children with uncomplicated malaria (n = 134) and black bars children with complicated malaria (n = 16). Mean MetHb levels were 1.57% (SD: 0.27) in healthy children. In non-complicated malaria it was 1.77% (SD:0.31) while it was 2.05% (SD:0.71) for complicated malaria; with both results significantly different from healthy children (p < 0.01). Each results represents the mean of triplicate measurements.

In Lambaréné, Gabon, a total of 282 children were included, 132 controls and 150 with malaria. The characteristics of the study population and the mean values for MetHb are shown in Table 2. The distribution of the MetHb values are represented in Figure 4. The mean value for healthy children was 1.57%. It was 1.77% and 2.05% in children with uncomplicated and complicated malaria, respectively, which was significantly different (p < 0.01) from the healthy controls, although the distributions show some overlap (Figure 4). The correlation coefficient for parasitaemia and MetHb was 0.09 (Additional file 3), while for MetHb and Hb it was -0.14 (Additional file 4).

**Table 2:** Characteristics of study population in Lambaréné, Gabon

	**(n)**	**Age, mean (SD)**	**Sex % (M/F)**	**Parasitaemia/μl, median (IQR)**	**MetHb %, mean (SD)**	**MetHb % (range)**
Controls	132	4.8 (0.7)	58%/42%	-	1.57 (0.27)	0.9-3.4
Uncomplicated malaria	134	4.4 (2.4)	54%/46%	14,214 (84,722)	1.77 (0.31)	0.6-2.8
Complicated malaria*	16	3.4 (1.9)	68%/32%	31,972 (41,300)	2.05 (0.71)	1.3-4.4

*Complicated malaria includes 5 children with cerebral malaria and 11 with severe anaemia.

## Discussion

The central chain of events in the haemolysis/MetHb/haem hypothesis [4,7,8] is based on the idea that levels of all relevant players, including free Hb, MetHb, liberated haem, HO-1 expression, CO and COHb ought to be increased in haemolytic conditions. Consequently, in diseases with acute major haemolysis, such as it occurs in a sickle cell crisis, one would expect to see markedly raised MetHb and COHb levels. In fact, a Tanzanian study reported that the highest COHb levels (6.6%) were seen in sickle cell disease [14]. The data from this study, obtained in Lisbon, are consistent with this (Figures 1, Additional file 1); which also confirms the notion that the clinical information obtained from the laboratory information system (LIS) was rather correctly and reflected the true clinical situation, further underlined by the fact that the highest COHb levels had the coherent clinical information of CO poisoning.

It should be noted that reference values can vary a lot, especially for COHb, from 1.5%-3% in non-smokers and up to 10% in smokers, highlighting the impact that environmental factors can have on these values [20-22]. Reference values for MetHb are more consistent, usually with an upper limit of 1%-1.5% [20,23,24]. However, a recent study reported that reference values for healthy children in Parana, Brazil were much higher (3.6%-6.4%) than for adults (1.9%-3.8%) illustrating the difficulty to find clear-cut values [25]. It thus appears doubtful if the measurement of COHb is a reliable way to assess haemolysis and disease severity.

The results obtained in Lisbon show that the distribution of infectious diseases, including sepsis, is similar to that seen in all other conditions, both for MetHb as well as COHb (Figures 1, Additional file 1). This contrast with reports where larger increases of CO levels were reported. One study found a median of 14 nmol/μl in 36 sepsis cases as compared to 7.3 nmol/μl in 21 non-sepsis cases and 2.1 nmol/μl in 12 HC when measured by gas chromatography in whole blood [26]. Another study from Indonesia found raised COHb levels in 20 severe sepsis cases (8%) as compared to 36 healthy controls (3.6%) [16]. Contrary to this, the results presented here appear to be more in line with a study of 5,322 ABGS measurements in 183 critically ill patients in New York, who reported a range of results from 0% - 4.8%; and which concluded that COHb levels did not seem to be clinically useful as marker of critical illness [27].

Furthermore, the recent study in 117 Indonesian adults also showed significantly raised COHb levels in moderately and severe malaria, with mean values of 7% and 10%, respectively, as compared to the 3.6% in healthy controls (HC). Interestingly, this contrasts with a much larger study in 1,520 Kenyan children [17]. Although they found increased COHb levels in malaria as well, with significantly different mean values in severe forms (4.3%) as compared to non-severe cases (4%), albeit the difference was much smaller with 0.3%. Surprisingly, all children who presented to hospital had raised COHb levels independent of the type of disease, with mean values ranging from 3.5% in burns to 6.6% in sickle cell disease, values which reportedly were not significantly different from malaria. In the study presented here no compelling differences for COHb were observed in the general patient population in Lisbon or the severe malaria cases in Berlin, the reason why this measurement was not performed in Gabon. Certainly, COHb levels may be influenced by many environmental factors, like smoke exposure or air-pollution [28], making them less suitable as a marker of haemolysis. Furthermore, although the non-invasive measurement with the RAD-57™ appears to detect alterations of COHb reasonably well [29,30], one study alerted that it may not be interchangeable with standard laboratory measurements [31].

### MetHb in malaria

As a baseline, in Lisbon MetHb levels were very similar between any infection and other clinical conditions, while only sickle cell disease showed a clear increase (Figure 2). Although some MetHb results were raised above 1.5%, only 98 samples (0.05% of all) were raised above 3%, with 11 samples having infections as clinical information.

Several studies have investigated MetHb levels in malaria, yet the results are often discrepant with regards to the absolute values as well as the magnitude of difference between the studied disease categories and HC. One study in 132 Tanzanian children reported significantly increased MetHb levels in uncomplicated and complicated malaria, with mean values ranging from 4.1%-5.8% as compared to HC with a mean MetHb level of 2%. However, no significant difference between the various forms of malaria were found [14]. In another study in adult Indonesians, median MetHb levels were only slightly increased with values of 0.8% in non-severe malaria and 1.2% in severe malaria as compared to 0.6% in HC [16]. The authors of this study suggest that different Hb levels in their study population as compared to the Tanzanian children might explain the discrepant observations. However, the mean Hb was 12.9 g/dL in the Indonesian HC and 11.1 g/dL in the Tanzanian HC, yet the MetHb levels observed in Indonesia were very different from those observed in Tanzania. On the other hand, in the Indonesian adults, Hb levels differed only by 2.3 g/dL between HC and patients with severe malaria, while in Tanzania the value was 4.7-6,5 g/dL lower in the three severe malaria groups as compared to HC. In fact, while the Tanzanian study reported a correlation of Hb and MetHb values, this was not observed in Indonesia.

In this present study, MetHb differed significantly between children with malaria (complicated or uncomplicated) as compared to HC. However the magnitude of this difference was modest, with some overlap of values in both categories (Figure 4). The correlation of MetHb with parasitaemia or anaemia was also rather low, although the Hb values ranged from 3 g/dL to 13 g/dL (Additional file 4). In the Berlin sub-study, no significant differences were observed between cerebral malaria *versus* other manifestations of severity, although the small sample size is clearly a limitation.

Thus, the results presented here appear to be more in line with two studies in Nigeria which reported more modest increases in MetHb levels in malaria: one study in 228 blood donors found a significantly higher MetHb level in *Plasmodium falciparum* positive individuals with a mean of 2.7% as compared to negative controls, with a mean of 2% [15] while another study in children showed raised MetHb levels in malaria (3.2%) as compared to no malaria (1.5%) [32].

The presented results still appear consistent with the haemolysis/methaemoglobin/haem hypothesis, and do not provide data to falsify this hypothesis [4]. Yet, the magnitude of the observed changes in MetHb levels are very discrete and observed values show a large overlap between groups (Figures 3 and 4). In fact, the difference is so discrete that one wonders if it is enough to serve as a convincing biological explanation for the pathogenesis in uncomplicated and complicated malaria according to this hypothesis. Interestingly, correlations between MetHb and Hb as well as parasitaemia were very low (Additional files 3 and 4). This could be explained by the fact that the Hb values rather reflected prior anaemia rather than an acute haemolytic event. Concerning parasites, it is more likely that MetHb levels reflect total parasite burden rather than parasitaemia, which is thought to be closer correlated with disease severity [33]. Still correlation coefficients in both situations were close to zero.

However, the non-invasive measurement was rapid and easy to perform and studies have confirmed that the method appears to be reliable as a screening tool for measuring MetHb and COHb [29,30,34]. Thus, apart from testing the haemolysis/methaemoglobin/haem hypothesis, further studies in different populations might be useful to assess if the non-invasive measurement of MetHb might not be an adjuvant tool for prognosis determination in resource-poor settings.

## Conclusions

Data from this study still appear to be consistent with the central chain of events in the haemolysis/MetHb/haem hypothesis, and they do not provide evidence to refute this hypothesis. However, the results show that MetHb and CO-Hb levels in infectious diseases, including sepsis, are similar to those seen in a broad range of other medical conditions, both for MetHb as well as COHb. With regard to malaria, although MetHb is significantly different between healthy control and uncomplicated or complicated cases in African children, the magnitude of the difference is very discrete. Nevertheless, the simplicity of the non-invasive measurement makes bedside MetHb testing a simple option. The current study was not designed to address if this measurement might be valuable adjuvant tool for prognosis determination in resource poor settings, an issue to be addressed in future studies.

## Abbreviations

ABGS: Arterial blood gas analysis; SD: Standard deviation; FBC: Full blood count; Hb: Haemoglobin; MetHb: Methaemoglobin; COHb: Carboxyhaemoglobin; CO: Carbon monoxide; NS: Statistically not significant (p > 0.05); HC: Healthy controls; LIS: Laboratory information system.

## Competing interests

The authors declare that they have no competing interests.

## Authors’ contributions

TH and MPG designed the study, analysed data and prepared the manuscript. TH collected the data in Lisbon. TZ collected the data in Berlin. TG, SL and AF collected the data in Lambaréné. JMC assisted in designing the study. All authors have contributed to and approved of the manuscript’s final version. All authors read and approved the final manuscript.

## Supplementary Material

Additional file 1 **COHb values in an unselected hospital population in Lisbon.** Distribution of Carboxyhaemoglobin values retrieved retrospectively from the laboratory information system for a one year period from an unselected population at the University Hospital, Lisbon (n = 17,720). Stratified by clinical information accompanying the request into major groups. Click here for file

Additional file 2 **COHb in patients with complicated malaria admitted to an Intensive Care Unit in Berlin.** Carboxyhaemoglobin values retrieved retrospectively from patients admitted with malaria to the Intensive Care Unit at the Charité University Hospital in Berlin (n = 13). All patients had criteria for complicated malaria as defined by the WHO, with eight patients having cerebral malaria (lines) and five patients having other criteria, as shown in Table 1 (dashed lines). Click here for file

Additional file 3 **Correlation of MetHb and parasitaemia levels in children with malaria in Lambaréné, Gabon.** Dot plot of MetHb (Y-axis) *versus* parasitaemia (parasites/μl) (x-axis). Click here for file

Additional file 4 **Correlation of MetHb and Hb levels in children with malaria in Lambaréné, Gabon.** Dot plot of MetHb (Y-axis) *versus* Hb (g/dL) (x-axis). Click here for file
