# Supplementary material for: Methaemoglobin and COHb in patients with malaria
Source: Malar J. 2014 Jul 23;13:285. doi: 10.1186/1475-2875-13-285 (PMC4118161; doi:10.1186/1475-2875-13-285)
Supplement: Additional file 1 — COHb values in an unselected hospital population in Lisbon. Distribution of Carboxyhaemoglobin values retrieved retrospectively from the laboratory information system for a one year period from an unselected population at the University Hospital, Lisbon (n = 17,720). Stratified by clinical information accompanying the request into major groups. [file 1475-2875-13-285-S1.pdf]

Additional File 1

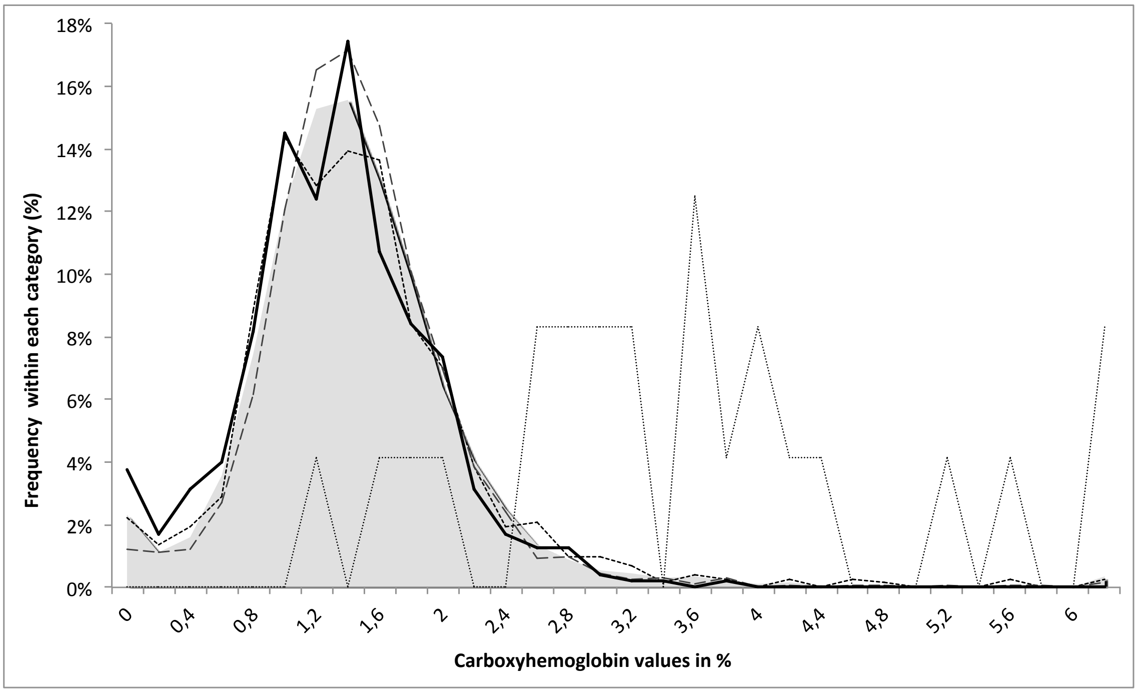

COHb values in an unselected hospital population in Lisbon

Distribution of Carboxyhemoglobin values retrieved retrospectively from the laboratory information system for a one year period from an unselected population at the University Hospital, Lisbon (n=17,720). Stratified by clinical information accompanying the request into major groups: shaded grey area – no infection (n=13948), solid black line – sepsis (n=476), dashed line - respiratory infection (n=2273), square doted line –other infections (n=725). As comparison for hemolytic condition: thin doted line – sickle cell disease (n=24). Histogram based on 0.1% intervals (bins) of COHb values. Y-axis represent percentage in each category. Note the absence of raised values in samples with clinical infection of infection, including sepsis. Samples with clinical information CO poisoning/inhalation (n=114) not included in analysis, because 38% show values >6% COHb.
