# Supplementary material for: Methaemoglobin and COHb in patients with malaria
Source: Malar J. 2014 Jul 23;13:285. doi: 10.1186/1475-2875-13-285 (PMC4118161; doi:10.1186/1475-2875-13-285)
Supplement: Additional file 2 — COHb in patients with complicated malaria admitted to an Intensive Care Unit in Berlin. Carboxyhaemoglobin values retrieved retrospectively from patients admitted with malaria to the Intensive Care Unit at the Charité University Hospital in Berlin (n = 13). All patients had criteria for complicated malaria as defined by the WHO, with eight patients having cerebral malaria (lines) and five patients having other criteria, as shown in Table 1 (dashed lines). [file 1475-2875-13-285-S2.pdf]

Additional File 2

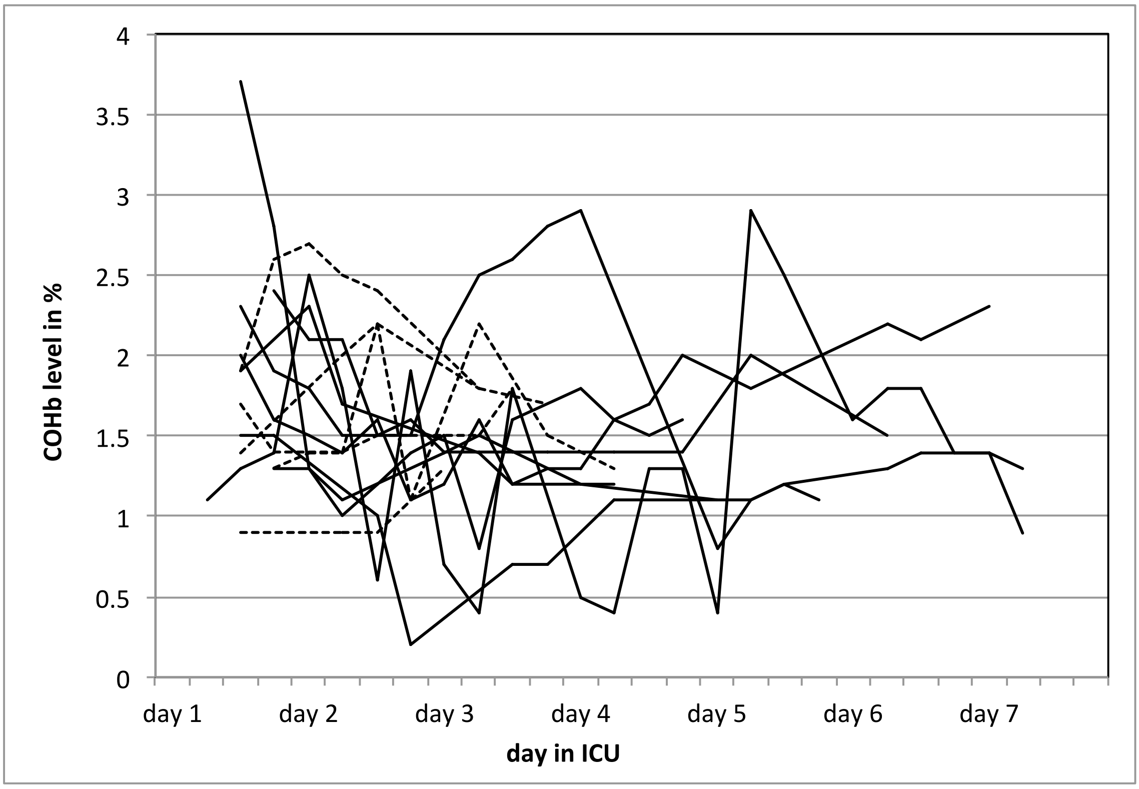

COHb in patients with complicated malaria admitted to an Intensive Care Unit in Berlin

Carboxyhemoglobin values retrieved retrospectively from patients admitted with malaria to the Intensive Care Unit at the Charité University Hospital in Berlin (n=13). All patients had criteria for complicated malaria as defined by the WHO, with 8 patients having cerebral malaria (lines) and 5 patients having other criteria, as shown in table 1 (dashed lines). All values are represented and lengths of the lines corresponds to time in the ICU. Mean value of COHb levels for the day on admission was 1.76% (SD: 0.62) for all measurements, and 1.90% (SD:0.63) for cerebral malaria while it was 1.46% (SD:0.57) for non-cerebral malaria (NS). Note that values during ICU stay may have been influenced by ventilator support and inhaled O2 concentration.
