# Supplementary material for: Methaemoglobin and COHb in patients with malaria
Source: Malar J. 2014 Jul 23;13:285. doi: 10.1186/1475-2875-13-285 (PMC4118161; doi:10.1186/1475-2875-13-285)
Supplement: Additional file 4 — Correlation of MetHb and Hb levels in children with malaria in Lambaréné, Gabon. Dot plot of MetHb (Y-axis) versus Hb (g/dL) (x-axis). [file 1475-2875-13-285-S4.pdf]

Additional file 4

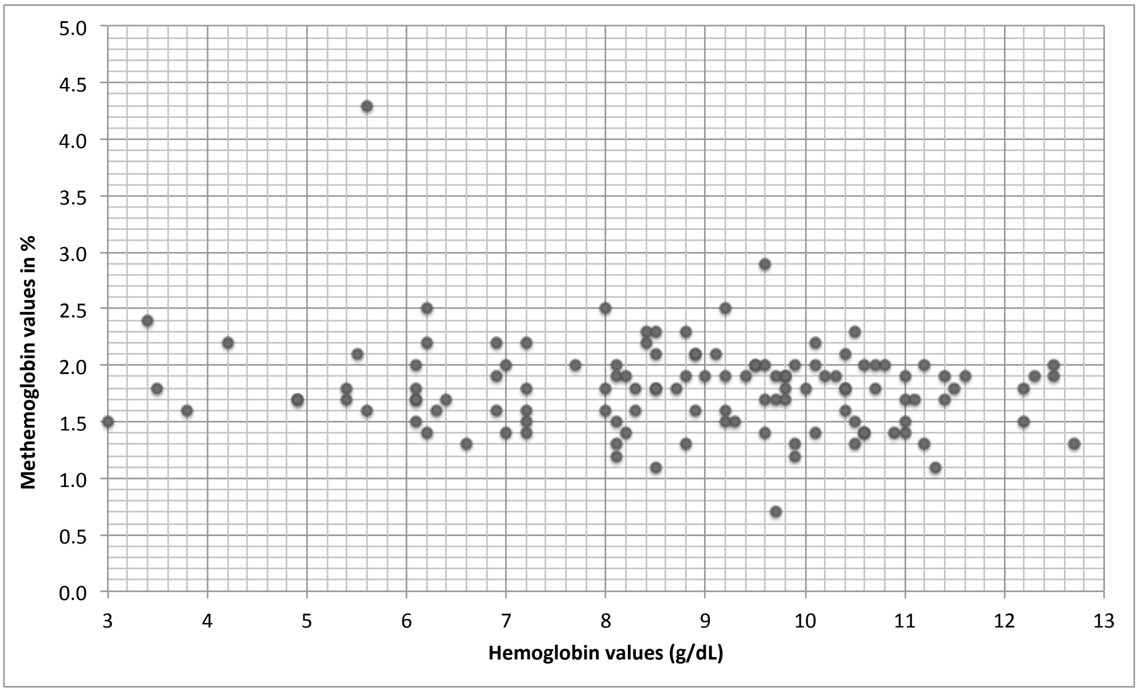

Correlation of MetHb and Hb levels in children with malaria in Lambaréné, Gabon

Dot plot of MetHb (Y-axis) versus Hb (g/dL) (x-axis). The correlation is r= -0.14 (P>0.1). MetHb levels were determined with the rainbow® pulse oximeter and Hb levels were determined using the ABX pentra 60 (Horiba Medical, Montpellier, France). Values were obtained only in malarious children, because they had a blood sample taken as part of their work up.
